# Advances in Omics Approaches for Abiotic Stress Tolerance in Tomato

**DOI:** 10.3390/biology8040090

**Published:** 2019-11-25

**Authors:** Juhi Chaudhary, Praveen Khatri, Pankaj Singla, Surbhi Kumawat, Anu Kumari, Vinaykumar R, Amit Vikram, Salesh Kumar Jindal, Hemant Kardile, Rahul Kumar, Humira Sonah, Rupesh Deshmukh

**Affiliations:** 1 Department of Biology, Oberlin College, Oberlin, OH 44074, USA; juhi.chaudhary@gmail.com; 2 National Agri-Food Biotechnology Institute (NABI), Mohali, Punjab 140306, India; p.khatri2712@gmail.com (P.K.); pankajsingla2614@gmail.com (P.S.); surbhikumawat002@gmail.com (S.K.); anuk991@gmail.com (A.K.); 3 Department of Vegetable Science, Dr. Yashwant Singh Parmar University of Horticulture and Forestry, Solan, Himachal Pradesh 173230, India; agrivinay123@gmail.com (V.R.); amitsolan@gmail.com (A.V.); 4 Department of Vegetable Science, Punjab Agricultural University, Ludhiana, Punjab 141004, India; saleshjindal@pau.edu; 5 Division of Crop Improvement, ICAR-Central Potato Research Institute (CPRI), Shimla, Himachal Pradesh 171001, India; kufrihemant@gmail.com; 6 Department of Plant Science, University of Hyderabad, Hyderabad 500046, India; Rksl@uohyd.ac.in

**Keywords:** proteomics, metabolomics, ionomics, genotyping by sequencing, genome-wide association study, quantitative trait loci

## Abstract

Tomato, one of the most important crops worldwide, has a high demand in the fresh fruit market and processed food industries. Despite having considerably high productivity, continuous supply as per the market demand is hard to achieve, mostly because of periodic losses occurring due to biotic as well as abiotic stresses. Although tomato is a temperate crop, it is grown in almost all the climatic zones because of widespread demand, which makes it challenge to adapt in diverse conditions. Development of tomato cultivars with enhanced abiotic stress tolerance is one of the most sustainable approaches for its successful production. In this regard, efforts are being made to understand the stress tolerance mechanism, gene discovery, and interaction of genetic and environmental factors. Several omics approaches, tools, and resources have already been developed for tomato growing. Modern sequencing technologies have greatly accelerated genomics and transcriptomics studies in tomato. These advancements facilitate Quantitative trait loci (QTL) mapping, genome-wide association studies (GWAS), and genomic selection (GS). However, limited efforts have been made in other omics branches like proteomics, metabolomics, and ionomics. Extensive cataloging of omics resources made here has highlighted the need for integration of omics approaches for efficient utilization of resources and a better understanding of the molecular mechanism. The information provided here will be helpful to understand the plant responses and the genetic regulatory networks involved in abiotic stress tolerance and efficient utilization of omics resources for tomato crop improvement.

## 1. Introduction

Tomato, one of the most valuable fruit and vegetable crops worldwide, is integral to the human diet. Due to the diverse range of its utility in raw, cooked, and processed food, the global demand for tomato has increased tremendously in recent years [1]. Although tomato is a temperate crop, it is being cultivated in diverse climatic zones, which makes the cultivation of tomato more challenging. Often, crop productivity and yield are severely affected by changing environmental conditions and abiotic stresses such as drought, salinity, and heat. Therefore, most of the non-conventional tomato cropping areas have adopted greenhouse-based cultivation for maintaining an uninterrupted supply throughout the year. Not only is the cost higher in greenhouse cultivation but there is also rapid accumulation of nitrate, phosphate, and salinity observed in the soil, which ultimately leads to soil degradation and groundwater or surface water pollution. Therefore, improvement of stress tolerance in tomato cultivars is sustainable and economically more desirable. Abiotic stress conditions imposed by extreme water and temperature regimes, nutritional imbalance in the soil substrate, elemental toxicity, and high salinity are the major factors limiting tomato production. The abiotic stresses become more complex under field conditions where more than one stressor typically coincide. Therefore, the development of sustainable, high-yielding varieties with improved tolerance to various abiotic stresses is a prerequisite to meet the global food demand [2]. Numerous efforts have been undertaken to address single stress traits under controlled conditions, but this approach is not always practical because the plant response is different in the field where multiple factors and stresses are imposed simultaneously [3]. In the past decade, conventional breeding has led to significant advances for a broad set of traits including biotic and abiotic stress tolerance, yield components and quality-related traits [4]. However, traditional varieties are susceptible to multiple stresses at different locations. Therefore, considering the genetic complexity and environmental interactions, application of more comprehensive and multidisciplinary approaches offers a better strategy to improve stress tolerance in modern crops [5,6,7]. 

Recent advances in the field of genomics have accelerated the successful development of new varieties. Molecular markers are based on the polymorphism identified in any given DNA sample, and they have dramatically increased the ability to characterize genetic diversity in the germplasm pool for essentially any crop species. Molecular markers have several advantages over the morphological or biochemical markers. These advantages include easy assay, reproducibility, convenience of use, high availability, stability regardless of environmental or external factors, and representation throughout entire genomes [8]. DNA markers have been widely used for versatile applications in genetics, molecular biology, genomics, and breeding in plants, including tomato. The most widely used applications of molecular markers in plant breeding include mapping of genes and quantitative trait loci, germplasm evaluation, population characterization, diversity studies, genomic fingerprinting, and marker-assisted breeding [9]. The efficiency of marker-assisted selection (MAS) for any trait during breeding requires precise information of map position and the molecular markers [10,11]. High-resolution mapping of QTLs, validation of linked markers, and marker conversion are the steps involved in the development of markers for MAS [12,13]. MAS has been extensively applied to the breeding of disease-resistant varieties in tomato [14]. MAS has been accelerated with the relatively recent adaptation of genotyping methods based on single nucleotide polymorphisms (SNPs) [15,16]. The availability of high-throughput marker genotyping systems and plentiful, well distributed markers make it possible to perform MAS more efficiently. In tomato, whole genome resequencing of 84 tomato accessions has identified over millions SNPs distributed throughout the entire genome [17]. Such a resource will be helpful for mapping studies as well as molecular biology research focusing on understanding the genetic regulation of different traits in tomato. 

**Figure 1:**
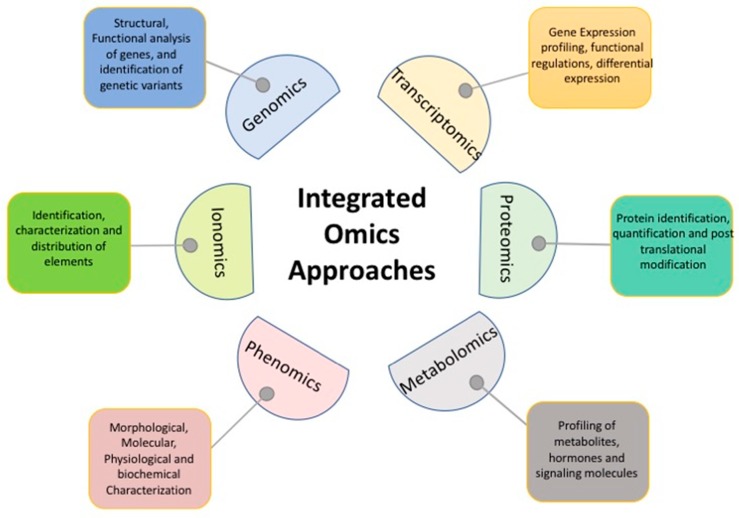
Different omics branches being used individually or in an integrated manner in plant science.

Recent technological advances have created several omics branches dealing specifically with the molecular components of cellular biology. To date, the major omics approaches include genomics, transcriptomics, proteomics, metabolomics, phenomics, and ionomics [5,18,19] (Figure 1). Omics approaches provide a holistic view of molecules at the cellular, tissues, or organism level. The integration of different omics providing many-dimensional biological information is being approached through a relatively new branch of life science known as system biology [20,21]. A recent development in DNA sequencing technology has accelerated genomics and transcriptomic research in plants and all other domains of life, including animals, fungi, and insects. Other omics branches like proteomics, metabolomics, and ionomics are not yet explored sufficiently as compared to genomics and transcriptomics. Since tomato has high economic importance and commercial value, it requires the integration of multi-disciplinary knowledge to design climate-smart varieties for high and stable yield in adverse climatic conditions. In this context, the present review provides in-depth information on recent advances in different omics branches, and methods for efficient exploration of available resources in tomato are discussed. The integration of different omics tools, techniques, and approaches to advance tomato research has also been addressed.

## 2. Tomato Genomics for Abiotic Stress Tolerance

### Whole Genome Sequencing and Resequencing

Sequencing of the entire genome is most efficient in accelerating molecular research. In plants, Arabidopsis was the first genome to be sequenced by an international consortium [22]. Its genome sequence helped in understanding genome organization, regulation, and evolution. Furthermore, with the invention of next-generation sequencing (NGS) technologies allowing parallel sequencing of millions of molecules simultaneously, whole genome sequencing became significantly cheaper and faster than the conventional methods [23]. Subsequently, many crop genomes, including tomato, have been sequenced using both Sanger’s and NGS methods [24,25,26]. The high quality, well-annotated tomato reference genome sequence is routinely used for genomics and transcriptomic studies. The more evident benefits acquired from genome sequencing include the catalog of annotated gene models, genome organization, syntenic information, repeats, and most importantly the basis to identify genetic variations (The Tomato Genome Consortium, 2012) [17]. The reference tomato genome also serves as the basis for the annotation of other Solanaceae species genomes.

With the availability of the high-quality reference genome, sequencing of the entire genome (resequencing) for many genotypes of the species is much easier and more cost-effective. Earlier efforts of resequencing eight tomato genotypes have identified more than 4 million SNPs, over a hundred thousand InDels, and seven thousand copy-number variations [27]. *Solanum pennellii* is known for its unique morphology as well as its extreme stress tolerance; therefore, it has been crossed with *S. lycopersicum* for the improvement of several agronomic traits. Bolger et al., 2014 performed high-quality genome sequencing of introgressed lines of *S. pennellii* x *S. lycopersicum* in order to identify the candidate genes associated with stress tolerance [28].

Furthermore, resequencing of two tomato landraces, COR and LUC, selected based on traits related to drought tolerance and fruit quality, identified hundreds of thousands of SNPs and hundreds of structural changes [29]. The sequence variation is expected to explain the high drought tolerance and adaptability to low water regimes in these genotypes. Further investigation of candidate genes identified about 122 genes with high effect SNPs (Non-Synonymous). Since both of the genotypes are drought tolerant, the genes with common variation have been selected as most promising candidates. The list of promising candidates includes heat shock proteins like Solyc05g055200, Solyc08g078720, Solyc09g011710, and Cation/H+ antiporters like Solyc03g032240, and Solyc09g010530 [29]. In addition, resequencing of plant pathogens also helps to understand co-evolution, particularly the rapidly evolving gene-for-gene system of virulence and resistance factors in the pathogen and host plant, respectively [30,31,32]. In recent years, pan-genome sequencing has become increasingly significant because it adds depth and completeness to the reference genome. Recently, genome sequences of 725 accessions were utilized for tomato pan-genome sequencing which revealed that 4873 genes were not identified in the reference genome. The study further performed presence/absence variation analyses in order to comprehend substantial gene loss and intense negative selection of genes and promoters during tomato domestication and improvement [33].

## 3. Molecular Markers Resources in Tomato

The whole genome sequence of tomato has been explored extensively for the development of molecular markers. Microsatellites or simple sequence repeats (SSRs) are one of the promising marker systems suitable for the labs where SNP genotyping is not feasible. Genome-wide identification of microsatellites and subsequent marker development creates a valuable resource for breeding programs [8].

With the advent of cost-efficient and high-throughput genotyping methods, SNP genotyping methods are gaining wide popularity. Among the several SNP-based genotyping methods, the genotyping by sequencing (GBS) approach is a highly multiplexed system for constructing RRL (reduced representation libraries), molecular marker discovery, and genotyping for crop improvement [34,35]. Due to low cost and advancing technologies, GBS has been applied to several crop species [36,37]. For example, a tomato GBS study led to the discovery of 8,784 SNPs based on an NGS approach. Of these SNPs, 88% are frequently observed in tomato germplasm [38]. 

Even though GBS is a simplified and cost-effective approach, its use is restricted because of the computational and data analysis expertise required. It may be widely used in the future with the development of computational packages and pipelines [16].

## 4. Identification of Loci Governing Abiotic Stress through QTL Mapping and GWAS

**Figure 2:**
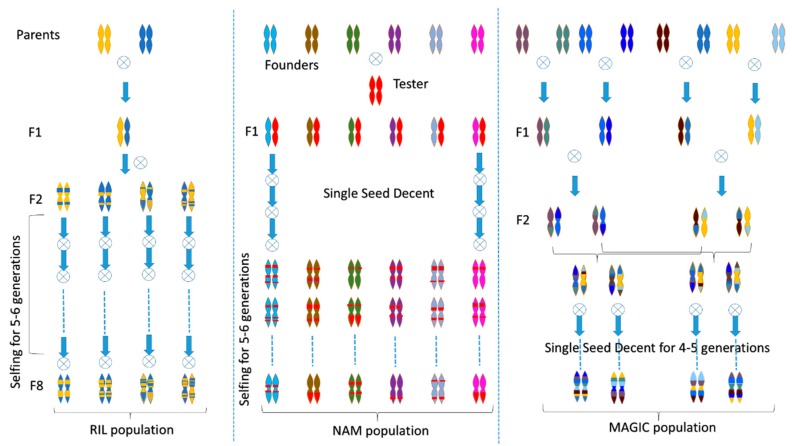
Examples of strategies for the development of conventionally used mapping population such as Recombinant inbred lines (RILs) and more current strategies such as nested association mapping (NAM) and multi-parent advanced generation inter-cross (MAGIC).

Genetic fingerprinting, linkage maps, and QTL mapping are marker-based approaches that require extensive genotype data. Linkage mapping and association mapping have led to the detection of QTL by identifying marker-trait associations [39]. A lot of focus has been given to mapping QTLs for several abiotic stresses such as salinity, drought, and low temperatures in tomato; however, other stresses like high temperatures, limited nutritional regimes, and environmental pollutants (heavy metals, ozone) still need to be explored. QTL mapping was performed using a linkage map of 1345 markers spaced at an average interval of 1.68 cM, representing 524 unique map positions. The genetic map covers more than 84% of the 900 Mb tomato genome and measures 2,156 cM [40]. The study identified QTLs regulating seed germination under different stresses. Several QTL mapping studies have been performed in tomato particularly to identify loci governing stress tolerance (Table 1). Similarly, genomics advances facilitated more complex approaches involving multi-parental populations like nested association mapping (NAM) and Multi-parent advanced generation inter-cross (MAGIC) (Figure 2). Up to now, very few studies exploring NAM and MAGIC populations in tomato have been published [40]. 

In contrast, a GWAS (Genome-wide association studies) approach has an advantage over linkage mapping as it explores the genetic diversity and recombination events present in germplasm collections and provides higher mapping resolution [18]. Therefore, GWAS has been routinely used to detect SNPs for agronomic traits in a world-wide tomato germplasm collection [45]. For instance, in tomato GWAS was performed using 182 SSR markers to identify the chromosome regions associated with 28 different volatile molecules defining tomato flavor [46]. Furthermore, GWAS studies have been done for fruit metabolic traits and other traits but there is no study of GWAS for abiotic stress in tomato yet. 

## 5. Genomic Selection (GS) for Abiotic Stress in Tomato 

The decreasing cost of SNP assays has made it possible to genotype large number of experimental lines allowing the implementation of the GS approach in crop breeding programs. The GS approach is efficient in simultaneously tracking all the loci contributing to trait development, irrespective of the magnitude of their individual effect. The GS approach overcomes the limitation of QTL mapping-based breeding where tracking/identification of small effect QTLs is difficult. Importantly, the small effect QTLs may collectively produce larger effects on economically important abiotic traits. [47]. Most economically important traits are complex and affected by unexpected trait expression because of epistatic interactions [48]. Therefore, GS is the best approach to predict genetic values for selection by utilizing all available molecular markers in combination with the phenotypic data of a training population. In this approach, a model that is used to establish and evaluate genotypic and phenotypic data to assess the phenotypic variation based on their whole genome genotypes (genetic composition) [19]. To determine breeding values, different GS algorithms like non-linear regressions (RKHS and RF), Bayesian approaches (Bayes A and B), and penalized regressions (RR, LASSO, and EN) have been developed. Among the available approaches, non-Linear regression is considered the best approach for prediction accuracies [49].

In tomato, GEBV (genomic estimated breeding value) is used majorly for yield and flavor improvement; fruit weight and SSC (soluble solid content) was calculated and gives the highest predictability in tomato [50]. Furthermore, tomato fruit quality was studied to analyze the accuracy of genomic selection for several metabolic and quality traits. In this study, GS has been performed for 45 phenotypic traits using a set of 163 tomato accessions as a training population (TNP). The overall conclusion was that several parameters such as the number and density of markers and the size of TNP affect the accuracy of prediction [51]. Overall, the use of high throughput phenotyping together with genomic information can help to enhance prediction accuracy and accelerate genetic gains by shortening the breeding cycle. Therefore, GS has a clear-cut advantage over MAS and association mapping for complex traits and notably contributing to the development and release of new cultivars. 

## 6. Advances in Transcriptomics

A wide range of environmental factors challenge plants, including tomato, for optimum growth and development. In response, the plant often activates defense mechanisms to mitigate adverse conditions. Understanding the gene regulatory cascades for such responses is very important for the effective management of abiotic stress. Therefore, collection and comparison of the transcriptome of different tissue types, and developmental stages is the best strategy to investigate plant response regulation and to identify genes involved in stress tolerance mechanisms. Thus, understanding the transcriptome of different tissue types or developmental stages would lead to a deeper understanding of corresponding phenotypic change [52]. Many tools and techniques are available for the evaluation of the transcriptomic data to get expression profiling for the gene-by-gene as well as collectively for many genes at a time [53]. 

**Figure 3:**
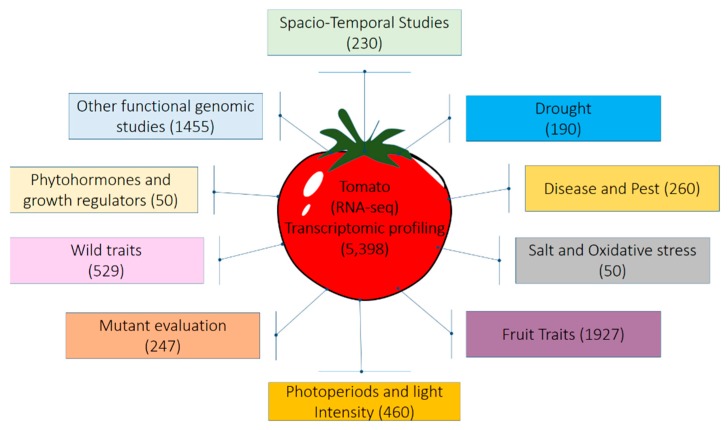
Transcriptomic resources generated through RNA-seq approaches in tomato being used for different studies. The values provided in the parenthesis indicate approximate number of RNA-seq sequenced libraries publicly available at SRA database (www.ncbi.nlm.nih.gov/sra).

Microarray has been used to identify the differentially expressed transcript of genes in response to abiotic stresses, including salinity and ABA, however very few studies have been undertaken for cold, drought, and oxidative stress [54,55]. Numerous studies have been conducted in tomato using transcriptomic approaches to identify genes having significant role in stress tolerance mechanisms (Table 2) as well as for the understanding of diverse molecular mechanisms (Figure 3). The Tomato Expression Database (TED) was developed (http://ted.bti.cornell.edu) which includes raw gene expression data derived from the public tomato cDNA microarray as well as experimental design and array information in compliance with the MIAME guidelines and provides web interfaces for researchers to retrieve data for their own analysis and use. In addition, the Tomato Digital Expression Database contains raw and normalized digital expression (EST abundance) data derived from analysis of the complete public tomato EST collection containing >150 000 ESTs derived from 27 different non-normalized EST libraries [56].

With the rapid development in next-generation sequencing, RNA sequencing (RNA-seq) has become the most cost-effective, efficient, and high throughput transcriptomic technology. Unlike microarray, the RNA-seq approach is not only confined to compare the transcripts levels, but it is also useful in novel gene discovery and spliced forms, especially in non-model plants. The impact of drought stress on gene expression has been analyzed with high-throughput transcriptomics in various plants such as rice [57], maize [58], and poplar [59]. In one of the studies, 400 drought responsive genes have been identified using microarray profiling of two drought tolerant tomato lines and a drought-sensitive cultivar (M82) [60]. The information generated with the microarray profiling will be helpful to identify regulatory genes and the molecular pathways involved in the drought tolerance mechanism in tomato.

Analysis of differential co-expression may also assist in investigating key regulatory steps in the metabolic pathway. Gene co-expression networks can be conveniently constructed using data generated by high-throughput gene expression profiling generated through RNA sequencing or microarray (Supplementary Figure S1). Co-expression analysis derived from the microarray and RNA-Seq data reveals that co-expression estimates are stable even when network constructed from mixed data of both. Furthermore, the tomato expression atlas (TEA; http://tea.solgenomics.net/) offers simultaneous visualization of groups of genes at a cell/tissue level of resolution within an organ in order to facilitate candidate gene identification [67]. Differential co-expression may assist to evaluate the regulatory steps in metabolic pathways. The gene co-expression, for instance, occurred in two metabolic pathways involved in lycopene and flavonoid biosynthesis [68]. In a recent study, graft-healing-related gene networks were identified. The study concluded that a large proportion of modules represented asymmetric expression networks from different pathways that were related to position. Additionally, auxin and sugar transport and signaling-related genes expression increased above the cut while stress response-related genes were found to be upregulated below the cut. The study concluded that some modules were related to graft union formation, among which oxidative detoxification genes were co-expressed along with both wounding response and cell wall organization genes [69]. Development in transcriptomics has driven the discovery of novel regulators in response to several abiotic stresses, for example, GLYCOALKALOID METABOLISM (GAME) 9, also called as JRE4, an AP2/ERF transcription factor, was identified in tomato as the main regulator of the SGA pathway [70,71,72]. Klee and Jiovannani (2011) elaborated the involvement of ethylene and the transcription factors with the ripening process and how the network controls the tomato fruit ripening and quality [73].

## 7. Tomato Proteomics: Applicability and Challenges

The proteomics approach commonly utilizes two-dimensional (2-D) gel electrophoresis, mass spectrometry (MS), matrix-assisted laser desorption ionization–time of flight (MALDI TOF), western blots, and ELISA in combination with bioinformatics tools to identify proteins and map their interactions in a cellular context. The precise quantification of proteins and peptides is challenging in fruit and root tissue, creating a significant bottleneck in proteomic studies. Nevertheless, MS-based methods in combination with computational tools are capable of processing hundreds of peptide transitions simultaneously as well as enabling good reproducibility, predictability, and accuracy. Furthermore, label-free quantitation procedures are convenient as well as provides reliable data for global protein expression studies; this method has been used extensively for assessment of tomato lines [74,75].

In tomato, a comparative proteome analysis was performed to study the impact of aluminum on cotyledon development of tomato [76]. Forty-nine proteins were differentially accumulated, affected by Al stress in tomato [76]. In another study, proteomic analysis of tomato seedlings subjected to Silicone and salt stress, a total of 40 differentially expressed proteins were identified using 2D-gel electrophoresis. Furthermore, twenty-four proteins found to be associated with the stress were up-regulated by Si supplements and down-regulated in salt-stressed root libraries [77]. A total of 52 differentially expressed proteins and many novel proteins were identified in tomato leaves in response to waterlogging stress. These proteins are involved with various processes such as photosynthesis, disease resistance, stress and defense mechanisms, energy and metabolism and protein biosynthesis [78]. Chilling tolerance with hot water treatment was studied in tomato fruits, the study concluded by using a hot water treatment to induce chilling injury tolerance. The chilling injury tolerance is thought to be due to the prevention of protein denaturation and activation of the antioxidant compounds [79]. Moreover, proteomic analysis for several stresses have been performed in tomato. Proteomics used for the identification of proteins responsive to NaCl and NaHCO3 stress [80], temperature stress [81], and drought tolerance [82]. Proteins related to temperature-related stress has been extensively in tomato, for example, 67 differentially expressed proteins were identified in tomato seedlings in response to high temperature using 2D-gel electrophoresis and MALDI-TOF/TOF MS ([83]. In a recent study, proteomic analysis was used to dissect the changes in contrasting tomato varieties under low-temperature stress [84]. Although a number of proteomics analyses have been performed to study abiotic stress response in tomato, data analysis and interpretation is still a bottleneck on the road of in-depth proteomics studies in crops. However, public databases with improved protein annotations are becoming available. The new proteomics advancements will help identify more regulatory candidate proteins and will ultimately direct to the development of stress-tolerant crops with higher yield and quality.

## 8. Tomato Metabolomics

Metabolomics is one of the promising approaches which provide a biochemical snapshot of an organism’s phenotype. Metabolomics allows the systematic identification and quantification of low-molecular-weight molecules that are closely associated with important toxicological and nutritional characteristics. Knowledge of genes, proteins, and transcriptomes are not enough to identify a cell completely, it is necessary to study the wide range of primary and secondary metabolites present in a cell. Numerous studies have been performed to understand the role of metabolites under high salinity and drought stress conditions in plants. Techniques such as GC-MS (Gas Chromatography-Mass Spectrometry) [85], CE-MS (capillary electrophoresis–Mass spectrometry [86] and NMR (nuclear magnetic resonance) [87] have been used to study metabolites for stress response in plants. In tomato, few studies have been performed for both biotic and abiotic stress responses, among them very few for abiotic stresses. 

Different drought-tolerant tomato varieties were studied to test the effects of water stress on flavonoids and caffeoyl derivatives. In five cultivars of cherry tomato varieties, water stress resulted in decreased shikimate and phenolic compounds [88]. Low oxygen stress can be induced by storage of fruit and vegetables under a closed atmosphere. In another study, the metabolic response of plant organs to low oxygen levels was examined and cultured tomato cells were used for the metabolic study to low oxygen. It was revealed that low oxygen stress altered the metabolic profile of tomato cells by accumulating the glycolysis intermediates in addition to increased lactate and sugar alcohols [89]. 

The integration of metabolomics, linkage mapping studies, and metabolome-based genome-wide association studies (mGWAS) provide comprehensive insight into the extent of natural variation in metabolism and its genetic and biochemical control in tomato [90]. Recently, Nunes-Nesi et al., 2019 conducted a study to identify leaf mQTL in tomato that are potentially important with respect to stress responses and plant physiology. The study identified 42 positive and 76 negative mQTL which are involved in the regulation of leaf primary carbon and nitrogen metabolism [91]. Indeed, metabolomic studies in tomato have increased our understanding of several metabolite networks and pathways related to many economic traits. The application of metabolomics to study abiotic stress will help us to elucidate underlying molecular mechanisms associated with stress and would surely lead to developing tolerant tomato plants with enhanced yield.

## 9. Tomato Ionomics

Ionomics is the study of the accumulation of essential and non-essential elements (metals, metalloids, and nonmetals). It can be applied to various types of plant species. There are various factors such as plant species, variety, organ, and environment which affect the ionome profile. Ionomics have been used to understand the role of mineral elements in plants by using high-throughput technologies like Inductively Coupled Plasma-Mass Spectrometry (ICP-MS) and ICP-Atomic Emission Spectrometry (ICP-AES) [92].

A wide range of studies have been done in the field of ionomics mainly on silicon (Si). Most of the dicots and particularly the Solanaceae family, take up small quantities of silicon and accumulate less than 0.5% in their tissue. Silicon has been found to improve drought tolerance and delay in wilting and benefit certain plants when they are under stress. In addition, many reports showed that the plants growing under heavy metal stress in the presence of silicon had reduced ROS (reactive oxygen species) contents which is indicative of enhanced stress response [93,94,95]. A study was conducted to investigate effect and mechanism of exogenous Si on salt tolerance in tomato, silicon was found to be responsible for the decreased concentration of Na and Cl in roots, stem, and leaves without any disturbance in translocation from root to shoot [96]. Furthermore, hydroponics tomato analysis revealed Si alleviated the effect of salinity stress on plant photosynthesis, chlorophyll concentration, and water content of leaf [97]. The fruit analysis of several tomato cultivars revealed that Si addition has a significant effect on arsenic uptake [98]. There are several elements in their different biochemical forms yet to be studied extensively enough to understand their precise role in plants. 

## 10. Phenomic Advances for Abiotic Stress Tolerance in Tomato

Phenomics is the study of high-throughput analysis of phenotypic variation which is a complex web of interactions between genotype, phenotype, and environment. The genome and phenome (a set of all phenotypes) studies performed with individuals or with large populations are complementary to each other [99]. The plants with tolerant phenotypes are good genomic resources and also become a target to identify the alleles using high throughput sequencing. Advances in sequencing technologies have improved genotyping efficiencies while phenotypic characterization has progressed more slowly in the past decade which limits the characterization of quantitative traits especially those related to stress tolerance [100]. However, there are developments in phenotyping methods which allows the identification of specific characteristic. The use of advanced imaging systems, sensors, automations, and computational resources for the phenotyping in plants make phenomics a high-throughput approach capable of handling thousands of genotypes for the evaluation of hundreds of phenotypic parameters simultaneously [100,101,102]. There are a number of phenomics platforms available such as scan analyzer 3D, used to investigate physiological parameters in tomato plants under drought conditions [103].

Due to the complications in phenomics data collection, it requires the collaboration between scientists from diverse area of expertise. In addition, phenomic data collection is expensive and time-consuming. Integrated technical advances would therefore aid to lower the associated costs and enhance phenomic throughput. 

## 11. Integration of Omics Technologies

The recent progress in omics approaches (genomics, transcriptomes, proteomics, metabolomics, ionomics, and phenomics) has generated a huge amount of data which can be used to identify novel genetic and chemical elements controlling various physiological processes [104]. However, using only one approach is not sufficient to understand the complexity of stress response in plants which requires the integration of various approaches to comprehend the complex stress response (Figure 1). In addition, the analysis of high throughput data from various omics approaches is one of the biggest challenges to interpreting the response mechanism(s). Although there is a range of software tools for basic data analysis to meet these omics data challenges, there is still the requirement of collaborative approaches to understand the complex physiological and biochemical responses under stress conditions. To develop novel climate-smart crop varieties, efficient integration of different tools, techniques, and approaches looks like a promising strategy [105,106]. 

Understanding the precise molecular function of genes is a challenging task for the plant molecular biologist. In this regard, the QTL mapping and GWAS approaches provide information of regulatory loci governing a particular trait which can be used further to identify candidate genes. The candidate gene identification reduces the molecular biology efforts by reducing the number of genes that need to be functionally evaluated. The choice of technique to identify the genomic loci is a crucial step. GWAS can be applied to any set of germplasm and detects regulatory loci for several traits simultaneously that show variation [107]. Both approaches, QTL mapping and GWAS identify a chromosomal region that is associated with a particular trait. QTL regions are quite large, and harboring too many genes makes it difficult to identify the candidate gene, but the combination of QTL and GWAS approaches has been successfully used to identify candidate genes in soybean [107]. Furthermore, the integration of RNA sequencing profiles with gene expression profiles can provide vital clues for the identification of functions of unknown genes [108,109]. Therefore, combining QTL and GWAS with transcriptome profiling will be helpful to identify differentially expressed candidate genes [110,111]. A study performed in tomato by integrating information of QTLs, eQTLs, and differentially expressed genes identified candidate genes regulating water stress tolerance [112].

Efficient adaptation of computational techniques by the plant breeder largely depends on features such as user-friendly interface, easy accessibility, online tutorials and manuals, and interactive options. In this regard, several user-friendly databases useful to integrate omics scale data from different approaches have been developed for tomato, as described in Table 3.

## 12. Conclusions

Abiotic stress is one of the major limiting factors in plant growth and yield. Various omics tools and techniques have been developed to understand the molecular mechanisms of plants responses in abiotic stress conditions. Under stress conditions, plants modulate themselves to adopt the existing stresses by controlling gene regulation, proteins, and metabolites. It is essential to elucidate the functions of newly identified stress-responsive genes to understand the abiotic stress responses of plants. To identify changes by various tools and techniques like genomics, transcriptomics, metabolomics, ionmics, and phenomics have been devised to allow the understanding of genetic makeup in depth, their signaling cascade, and their adaptability under stress conditions. In tomato, genomics, ionomics, and transcriptomics have been developed for abiotic stress, but the other major branches like metabolomics, proteomics, and phenomics are as of yet lingering behind. Diverse study of omics tools and integrated approaches discussed in this review tell us about the current situations and future points for successful management of abiotic stress in tomato.

## Figures and Tables

**Table 1:** Significant quantitative trait loci (QTL) mapping studies performed to identify loci governing abiotic stress tolerance in Tomato.

Sr.No.	Stress	Trait	QTL	Chromosome	Position (cM)	LOD Score	R (%)	References
1	Cold Tolerance	RGR (Relative Germination Ratio)	qRGI-1-1	1	40.4	5.45	19.55	[41]
			qRGI-1-2	1	47.2	2.53	8.52	
			qRGI-4-1	4	10.4	2.06	6.02	
			qRGI-9-1	9	7.8	2.12	5.95	
			qRGI-12-1	12	8	4.26	11.33	
		CI (Chilling index)	qCI-1-1	1	9.8	3.25	0.95	
			qCI-2-1	2	18	2.96	10.34	
			qCI-3-1	3	0	3.01	10.31	
			qCI-9-1	9	26.8	2.35	7.31	
2	cold stress	Seed Germination	cld1.1	1	7	7.41	30.95	[42]
			cld1.2	1	8.8	4.27	17.24	
			cld1.3	1	15.4	2.27	9.78	
			cld4.1	4	13.8	2.06	9.13	
			cld4.1	4	20.7	2.98	13.15	
			cld8.1	8	2.5	1.26	5.76	
3	Salt Stress	Seed Germination	slt1.1	1	7	2.66	10.86	
			slt1.2	1	8.8	3.53	13.58	
			slt2.1	2	18.7	1.2	6.4	
			slt5.1	5	16	1.52	8.32	
			slt7.1	7	4.5	1.52	7.16	
			slt9.1	9	21.4	2.01	6.3	
			slt12.1	12	7.1	2.4	12.41	
4	Salt Tolerance	Seedling Stage	Stlq4	4			63.6	[43]
			Stlq6	6			64.8	
			Stlq9a	9			61	
			Stlq9b	9			63.6	
			Stlq12a	12			63.3	
			Stlq12a	12			61	
			Stlq12b	12			64.4	
5	Heat tolerance	Pollen viability	qPV11	11	19.4		36.3	[44]
		Pollen Number	qPN7	7	134.7		18.6	
		Style protrusion	qSP1	1	16		19.5	
			qSP3	3	80.4		28	
		Anther length	qAL1	1	70		15.5	
			qAL2	2	80.8		11.6	
			qAL7	7	134.7		25.2	
		Style length	qSL1	1	16		22.7	
			qSL2	2	80.8		10.5	
			qSL3	3	75.8		15.8	
		Flowers per	qFPI1	1	40		38.7	
		inflorescence						
		Inflorescence number	qIN1	1	39		21.9	
			qIN8	8	95.3		13.4	

**Table 2:** Major Transcriptomic analysis for abiotic stress tolerance in tomato.

Trait	Platform	DEG	Key Point	References
Microarray gene expression data of tomato to study meta-analysis of stress response	Affymetrix tomato Genome Array	835	Expression profile of different genes under different conditions, Meta-analysis to characterize the candidate genes for abiotic stress.	[61]
Temporal stage of fruit development To study the transcriptome profiling of ERF family genes.	Tomato Gene Chip arrays	57	Over expression of ERF family genes in tomato has been shown to confer increased resistance to abiotic stresses.	[62]
Tomato leaf responses to exogenous ABA	Illumina RNA-sequencing	2787	Exogenous ABA has potential to up- regulate many genes related to stress tolerance.	[63]
*Solanum lycopersicum* cultivars (WT) and MT (Micro-Tom)	RNA-sequencing	619	BR-deficient (Brassinosteroids) Micro-Tom showed lower drought and osmotic stress tolerance. BR signaling is tightly connected with gene networks related to abiotic stress and development	[64]
Micro-Tom seedling	RNA sequencing IlluminaGAIIx Platform	6643	Salt and oxidative stresses regulate tomato cytokinin level and transcriptomic responses.	[65]
Different stages of cultivated and wild tomato (Root, stem, leaf, flower, fruit and seedling)	RNA sequencing Illumina high-throughput sequencing	Upregulated- 126 Downregulated-87	These DEG associated with salt resistance, drought resistance and fruit nutrition.	[66]

**Table 3:** Online databases developed for tomato for integrated omics.

Sr.No	Database	URL	Description/Applications	References
**1.**	KaFtom	www.pgb.kazusa.or.jp	Database for Micro Tom full length cDNA clones, Full length cDNA libraries for EST sequencing.	[113]
**2.**	MiBASE	http://omictools.com	Database for Micro Tom ESTs and tomato Unigenes EST Sequencing, ESTAnnotations, SNPs, SSRs, Gene ontology, Metabolic pathways of Gene expressions And Sequence similarities.	[114]
**3.**	Tomatoma (Micro Tom Database)	http://tomatoma.nbrp.jp	Micro Tom mutant Resources, Metabolite information, Phenotype information, TILLING.	[115]
**4.**	Tomatomics	http://omictool.com/tomatomics-tool	Full length mRNA sequences, Gene structures, Expression Profiles and functional annotations of genes.	[116]
**5.**	TGRD (Tomato Genomic Resources Database)	http://omictool.com/trgd-tool	Interactive browsing of tomato genes, micro RNAs, simple sequence repeats (SSRs), important quantitative trait loci.	[117]
**6.**	TFGD (Tomato Functional Genomic Database)	http://ted.bti.cornell.edu	Microarray Expression Database, Metabolite profile Data analysis, RNA Seq. Data	[118]
**7.**	KaTomics DB (Kazusa Tomato Genomic Database)	www.kazusa.or.jp	Database for DNA markers, SNP annotations, and genome sequences	[119]
**8.**	MoToDB Metabolome Database	http://appliedbioinformatics.wur.nl/moto/	LC-MS (Liquid Chromatography Mass Spectrometry)	[120]
**9.**	CoxPathDB	http://cox-path-db.kazusa.or.jp/tomato	To predict function of tomato genes from result of functional enrichment analyses of co-expressed genes.	[121]
**10.**	Sol Genomics Network	http://solgenomics.net	Browse the tomato genome, Find the sequence similarity, and Download annotations.	[122]

## Supplementary Materials

The following are available online at https://www.mdpi.com/2079-7737/8/4/90/s1, Figure S1: Co-expression correlation network of tomato genes developed using an online tool ATTED-II (http://atted.jp/).
