# Supplementary material for: Advances in Omics Approaches for Abiotic Stress Tolerance in Tomato
Source: Biology (Basel). 2019 Nov 25;8(4):90. doi: 10.3390/biology8040090 (PMC6956103; doi:10.3390/biology8040090)
Supplement: Supplementary file 1 [file biology-08-00090-s001.pdf]

biology

Supplementary Materials:

Advances in Omics Approaches for Abiotic Stress Tolerance in Tomato

Juhi Chaudhary, Praveen Khatri, Pankaj Singla, Surbhi Kumawat, Anu Kumari, Vinaykumar R, Amit Vikram, Salesh Kumar Jindal, Hemant Kardile, Rahul Kumar, Humira Sonah, and Rupesh Deshmukh

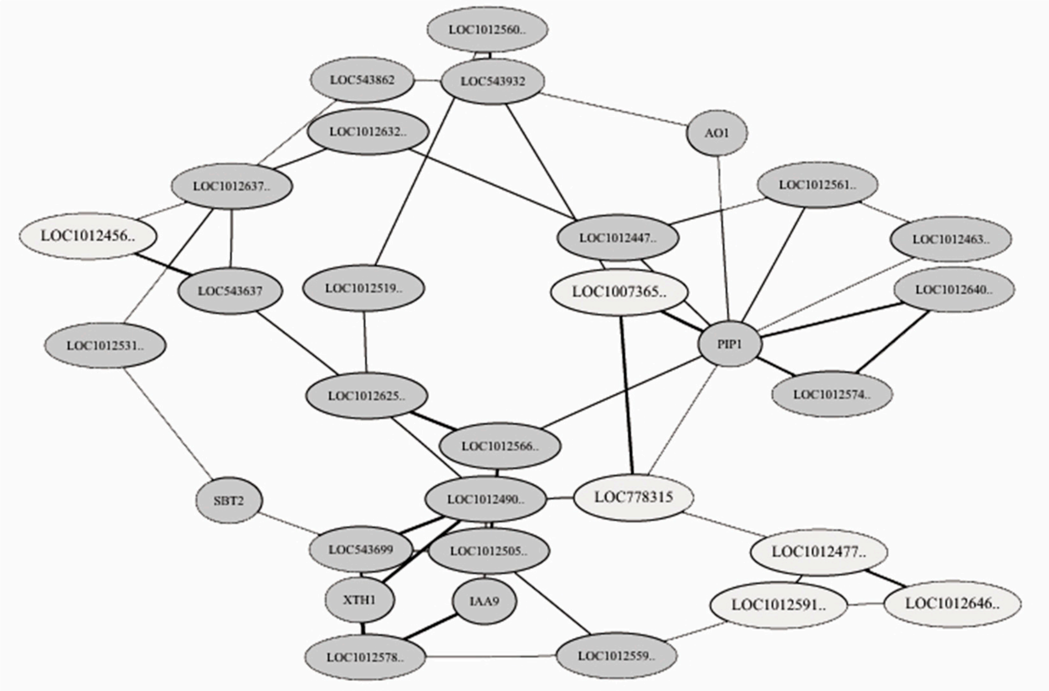

Supplementary Figure S1. Co-expression correlation network of tomato genes developed using an online tool ATTED-II (http://atted.jp/). The tool involves expression data of 401 microarray-based sample and 282 RNA-seq based samples. The figure depicts the correlation of Plasma Membrane Intrinsic Protein with other genes governing basic physiological function in tomato.
